# Copigmentation of Malvidin-3-*O*-Monoglucoside by Oenological Tannins: Incidence on Wine Model Color in Function of Botanical Origin, pH and Ethanol Content

**DOI:** 10.3390/molecules24081448

**Published:** 2019-04-12

**Authors:** Adeline Vignault, Jordi Gombau, Olga Pascual, Michael Jourdes, Virginie Moine, Joan Miquel Canals, Fernando Zamora, Pierre-Louis Teissedre

**Affiliations:** 1 Université de Bordeaux, Unité de Recherche Œnologie, EA 4577, USC 1366 INRA, ISVV, 33882 Villenave d’Ornon CEDEX, France; adeline.vignault@gmail.com (A.V.); michael.jourdes@u-bordeaux.fr (M.J.); 2 INRA, ISVV, USC 1366 Œnologie, F-33140 Villenave d’Ornon, France; 3 Departament de Bioquímica i Biotecnología, Facultat d’Enologia de Tarragona, Universitat Rovira i Virgili, C/Marcel.li Domingo 1, 43007 Tarragona, Spain; jordi.gombau@urv.cat (J.G.); olgapges@gmail.com (O.P.); jmcanals@urv.cat (J.M.C.); fernando.zamora@urv.cat (F.Z.); 4 Laffort, 11 rue Aristide Bergès, 33270 Floirac, France; virginie.moine@laffort.com

**Keywords:** oenological tannins, copigmentation, malvidin-3-*O*-monoglucoside, botanical origin, pH, ethanol content

## Abstract

The effect of the botanical origin, pH level and ethanol content of different oenological tannins on the color of malvidin-3-*O*-monoglucoside solution, including their effectiveness as copigments, was studied. Briefly, a model wine solution (4 g/L of tartaric acid, pH 3.5 and 12% ethanol) containing 50 mg/L of malvidin-3-*O*-monoglucoside was prepared and supplemented with 0.1, 0.2 and 0.4 g/L of commercial tannins using (−)-epicatechin as reference control copigment. Six additional model wine solutions (12% ethanol at pH 3.1, 3.5 or 3.9, and 10%, 12% or 14% ethanol at pH 3.5) were prepared as previously described. Samples were stored under airtight conditions. After a week the full absorbance spectrum in the visible range (400–800 nm) was measured and CIELAB color space was determined. These measurements, including an increase in a* (redness), a decrease in b* (yellowness) and a decrease in L* (lightness), indicated that all oenological tannins had a clear positive effect on color copigmentation. Moreover, hydrolysable tannins appeared to be better copigments than condensed tannins as the copigmentation effectiveness (Cp) was found to be between two to four times higher. The effects of these tannins were dose-dependent because a higher addition resulted in a greater impact on copigmentation. In general, an increase in pH and ethanol content resulted in a decrease of the effect of tannins on color. Independent of intrinsic wine conditions, hydrolysable tannins, more specifically gallotannin, remain the most effective in increasing red wine color. These results prove that supplementation with oenological tannins, especially hydrolysable tannins, could be an interesting tool for the improvement of the red wine color.

## 1. Introduction

Anthocyanins, are the main compounds responsible for the color of red wines. They are the major natural pigments in red wines, reaching typically 500 mg/L in young red wines [1], but their concentration depends on the grape variety and the growing conditions [2]. In red wines from *Vitus vinifera* grapes, the main monomeric anthocyanins are the malvidin-3-*O*-glucoside, cyanidin-3-*O*-glucoside, delphinidin-3-*O*-glucoside, pelargodinin-3-*O*-glucoside, peonidin-3-*O*-glucoside and petunidin-3-*O*-glucoside [3,4]. These anthocyanins are also present in acylated forms with acetic, coumaric and caffeic acids [5]. More derivatives of anthocyanins can be extracted or formed during the fermentation process [6]. The extraction and formation of new pigments or copigment complexes permit the color stabilization of red wine during the aging process since the monomeric anthocyanin content decreases constantly. Different phenomena can occur naturally to stabilize the color in red wines. Anthocyanins can form association structures with other colorless compounds (intermolecular copigmentation), with other anthocyanins (self-association), or in the case of coumaroylated and caffeoylated anthocyanins, the acylated esterified group can associate with the pyrylium ring of the anthocyanidin (intramolecular copigmentation) [7,8]. The copigmentation phenomenon is characterized by the formation of a “sandwich complex,” meaning a hydrophobic interaction (π–π stacking) between the B ring of the anthocyanin and the copigment (phenolic compounds, for example) [7]. These complexes adopt a sandwich-like structure that protects the flavylium cation against nucleophilic attack by water, avoiding the formation of the colorless hemiketal [9]. Consequently, copigmentation increases wine color intensity (hyperchromic effect), but it can also change the color hue through a bathochromic shift [10]. Finally, the formation of new pigments can occur, providing a large variety of pigments such as pyranoanthocyanins and polymeric anthocyanins [5].

The mechanism of pigment formation or copigmentation differs with respect to the botanical origins of oenological tannins. The term ‘tannins’ includes hydrolysable tannins from nut galls, tara, oak and chestnut, and condensed tannins from grape seeds and skins or other plant sources such as quebracho, mimosa and acacia [11]. Hydrolysable tannins are classified into two subfamilies: gallotannins and ellagitannins. Gallotannins are polymers formed by esterification between d-glucose and gallic acid. Ellagitannins are polymers of ellagic, gallic and/or hexahydroxidiphenic acids [11]. A nonahydroxyterphenoyl unit (NHTP) is esterified in positions 2, 3 and 5 with a C-glycosidic bond, while an open-chain of glucose is esterified in positions 4 and 6 with a hexahydroxydiphenoyl unit (HHDP), forming the chemical structure of ellagitannins [12]. Condensed tannins are polymers of flavan-3-ol units, differing mainly by the monomer released after acidic cleavage, the degree of polymerization (mDP), and their levels of galloylation and ramification [11]. They are also known as proanthocyanidins, because they release anthocyanins by acidic cleavage in accordance with the Bate-Smith reaction. Grape-seed tannins are composed only of procyanidins, while grape-skin tannins are composed of procyanidins and prodelphinidins. In addition, grape-skin proanthocyanidins have a higher mean degree of polymerization (mDP) and a lower level of galloylation than grape-seed proanthocyanidins [13]. In contrast, quebracho tannins are profisetinidins because their acidic cleavage yields fisetinidin and they have a high level of ramification, while mimosa tannins are prorobinetidins because they release robinetinidin [14]. Less is known about acacia tannins, but they appear to be composed of a mixture of profisetinidins, prorobinetidins and prodelphinidins [15]. In this way, condensed tannins can combine directly or indirectly with anthocyanins while hydrolysable tannins cannot participate in condensation reactions with anthocyanins. However, hydrolysable tannins, can participate in copigmentation reactions, as well as protect wine anthocyanins from oxidation since they may regulate oxidation-reduction phenomena [16]. Furthermore, the color stabilization of red wine depends not only on the anthocyanin content and the presence of copigment or yeast by-products, but also on the wine conditions such as pH level and ethanol content [17]. 

Currently, various commercial tannins are available on the market, differing in their chemical structure, botanical origin and preparation process. The use of these tannins to facilitate the finning of musts and wines was only recently authorized by the International Organization of Vine and Wine (OIV) [18]. Nonetheless, it is unquestionable whether oenological tannins can also be used for other purposes. Among the functions attributed to oenological tannins, their enhancing effect on color and stability of red wines is probably one of the main reasons of their wide use in current winemaking. Red wine color plays a very important role for consumers in their acceptance and perception of the product [19]. For this reason, winemakers are interested in gaining a better understanding of the role played by polyphenols, and more particularly, the interactions between tannins/anthocyanins to produce deeply colored wines with great color stability during aging [19]. To achieve this goal, winemakers apply different oenological practices, for example, saigné or thermovinification [20], addition of pectinolytic enzymes [21] and oak aging. Despite the various tools available to winemakers, none of these treatments have provided clear results without secondary problems or effects. The addition of oenological tannins has been proposed in the past, but due to the abundance of varieties that exist, their effects are not so well studied and clear. 

For these reasons, the aim of this study was to give standards in terms of co-pigmentation proportion and level for the major anthocyanin of wine, malvidine-3-glucoside. Additionally, this work aimed to verify and confirm the effectiveness of different types (botanical origin) of oenological tannins on wine color stability at different pH and ethanol contents to be applied as a new tool by winemakers when using tannins during winemaking or ageing. 

## 2. Results

### 2.1. Influence of the Botanical Origin of Oenological Tannins

**Figure 1:**
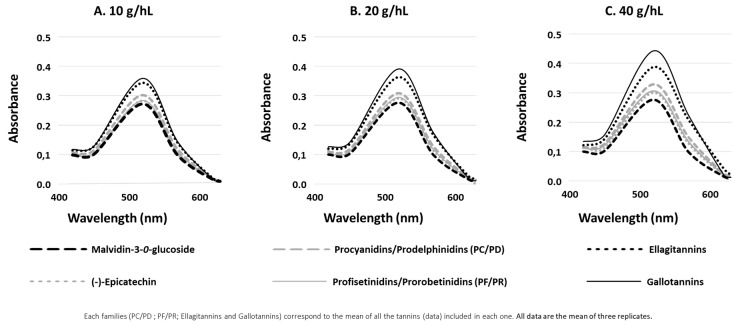
Visible spectra of model wine solution of malvidin-3-*O*-glucoside (50 mg/L) supplemented with 10 (**A**), 20 (**B**) or 40 (**C**) g/hL of (−)-epicatechin and oenological tannins after one week of experimentation.

**Figure 2:**
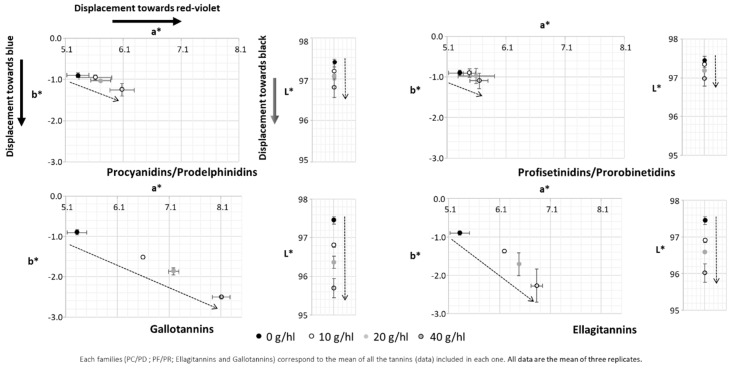
CIELAB color space of model wine solution of malvidin-3-*O*-glucoside (50 mg/L) supplemented by increasing concentration of (−)-epicatechin and oenological tannins after one week of experimentation.

**Figure 3:**
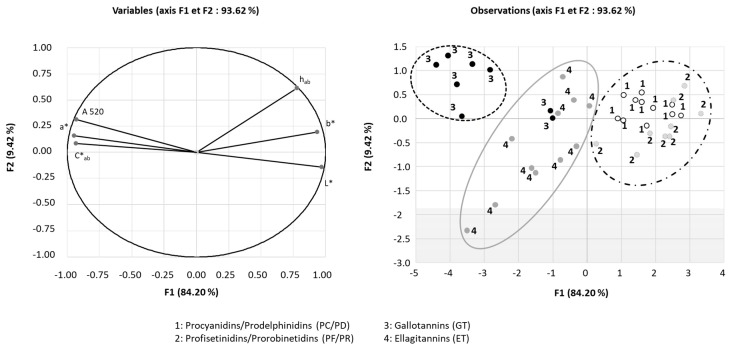
Plot of principal components analysis of model wine solutions of malvidin-3-*O*-glucoside (50 mg/L) supplemented by increasing concentrations of (−)-epicatechin and oenological tannins after one week of storage.

Table 1 displays the results obtained for each one of the 36 oenological tannins and (−)-epicatechin. In the case of the parameter b*, we observed a significant difference between grape-skin and grape-seed tannins and between acacia and quebracho tannins. Significant differences for the parameter L* were also observed between tannins within the same family in the case of the nut gall tannins and tara. Some differences can be noted between tannins from the same botanical origin, but in the majority of the cases, no significant differences were noted between tannins of the same family. In all cases, significant differences were noted between the different families of tannins. In fact, some significant differences were even noted between two tannins from the same botanical origin, but the differences between families were more revealing. Actually, in the case of vinification, a winemaker chooses the tannins according to their family, since in most cases, the equipment to test the purity of each tannin is not available. Thus, to better visualize and facilitate the understanding of the following figures (Figure 1, Figure 2 and Figure 3), tannins were grouped according to their family of botanical origin. Procyanidins/prodelphinidins (PC/PD) include tannins from grapes, grape seeds and grape skins, while profisetinidins/prorobinetidins (PF/PR) include tannins from acacia and quebracho. Gallotannins (GT) are comprised of tannins from nut galls and tara, while ellagitannins (ET) are comprised of tannins from oak and chestnut.

The full spectra within the visible range (400–800 nm) of the control sample (malvidin-3-*O*-glucoside) and solutions containing the different doses of the 36 oenological tannins and (−)-epicatechin after one week of experimentation are presented in Figure 1. In all cases, the presence of copigments brought about an increase in the absorbance at visible range, an effect which was dose-dependent. The higher the dose, the higher the absorbance of each compound (Figure 1A–C). This increment and dose-dependence are more evident in the case of hydrolysable tannins (gallotannins and ellagitannins). Hydrolysable tannins reached higher absorbance values (between A520nm = 0.35 to 0.45) than condensed tannins (between A520nm = 0.27 to 0.32) in all the cases. In the case of the highest dose (40 g/hL), gallotannins had an A520nm one and a half times higher than condensed tannins. Since the spectrum view only gives an idea of the color, CIELAB color space were measured to better understand this phenomenon. Lightness (L*) and Chroma (C*_ab_) describe quantitative attributes of the color, whereas hue (h_ab_) describe qualitative attributes [22]. The evaluation of these parameters makes it possible to determine the hyperchromic and bathochromic effects produced by the presence of the various copigments.

The red-greenness (a*), yellow-blueness (b*) and lightness (L*) components of the solutions of malvidin-3-*O*-glucoside enriched with increasing concentrations of (−)-epicatechin and oenological tannins are displayed in Figure 2. Positive values of a* are in the direction of redness and negative values in the direction of the greenness. Positive values of b* are in direction of ‘yellowness’, and negative for ‘blueness’. Finally, low values in L* point towards black while high values point towards white [19]. In general, all tannins yielded a positive effect as copigments and their effect on the color was dose-dependent since the higher the dose, the higher the resulting displacement. Clear differences in effects were observed between oenological tannins. GT was found to have the highest effect followed in decreasing order by ET, PC/PD and PF/PR. Condensed tannins (PC/PD and PF/PR) presented weak displacement, even though a higher displacement towards red was observed. Moreover, GT and ET presented similar displacement towards blue (b*) and black (L*) while GT presented higher displacement towards red (a*). The incrementation of the a* value was around 1.5 points in the case of ET versus 3 points for GT. Finally, in the cases of GT and ET, the dose-dependence seemed to be linear with a constant increment of the CIELAB color space according to the increment of the dose employed. These results confirm those obtained from absorbance measurements in Figure 1, indicating that hydrolysable tannins, especially gallotannins, are most effective as copigments because their hyperchromic (decrease of L*) and bathochromic (decrease of b*) effects are considerably higher. To better visualize the results obtained in Figure 1 and Figure 2, a plot of principal components analysis (PCA) was constructed by regrouping all the color parameters evaluated (A520nm, a*, b*, h_ab_, C*_ab_ and L*). 

PCA was performed on the variables and the observations of model wine solutions of malvidin-3-*O*-glucoside supplemented with increasing concentrations of (−)-epicatechin and oenological tannins, the results of which are displayed in Figure 3. Three different clusters are clearly visible: the first containing samples 1 and 2 corresponding to condensed tannins (PC/PD and PF/PR), the second comprised mainly of sample 4 (ET), and the third containing sample 3 (GT). The first cluster is placed to the right as the variables b*, L* and h_ab_, indicating that condensed tannins induce a displacement toward yellow, weaker color and provide a low impact on color stabilization. Conversely, because the third cluster is placed to the top left as the variable A520nm, a*, C*_ab_ and at the opposite of L* and b*, it can be concluded that GT induce a displacement toward red, blue and a darkness color meaning high color stabilization. The second cluster is placed to the left, but closer to the center of the PCA, meaning displacement towards red and blue, but to a lesser extent than GT. These results confirm that the botanical origin of oenological tannins influence their effectiveness regarding color stabilization and that gallotannins are the most influential, followed by ellagitannins and condensed tannins. 

Various authors have commented on the influence of the intrinsic conditions of red wine, specifically pH level [23,24] and ethanol content [17,25,26], on color stabilization. For this purpose, studies with five of the thirty-six oenological tannins (to represent the different botanical origins) at different pH levels and ethanol content have been performed to confirm the previous results (Figure 1, Figure 2 and Figure 3).

### 2.2. Influence of Copigment/Pigment Ratio and pH

**Figure 4:**
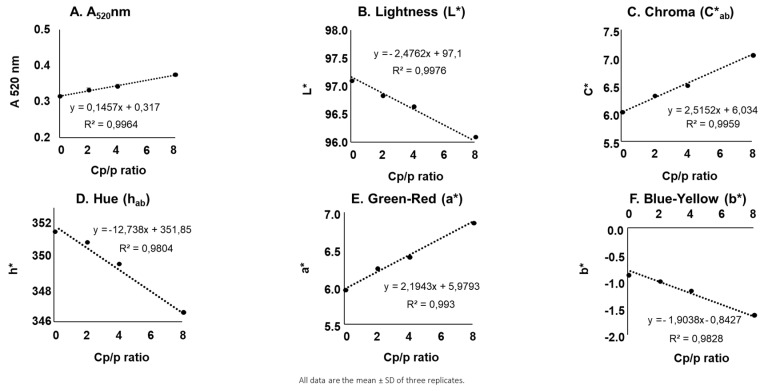
Example (case of ellagitannins at pH 3.5 and 12% of ethanol) of the determination of the Cp by the representation of A520nm (**A**) and CIELAB color space (**B**–**F**) versus copigment/pigment ratio of model wine solution of malvidin-3-*O*-glucoside (50 mg/L) supplemented by (−)-epicatechin or oenological tannins.

Figure 4 shows an example (pH 3.5 with ellagitannin) of the plots of A520nm and CIELAB color space versus copigment/pigment ratio for all oenological tannins and (−)-epicatechin. These plots allowed us to determine the copigmentation effectiveness (Cp), since the slope of the straight line indicates the extent to which copigmentation occurs according to the copigment/pigment ratio. The values of the slopes indicate the effectiveness of (−)-epicatechin and oenological tannins in improving the color. For A520nm, C*_ab_ and a*, a higher Cp indicates a greater impact while for b*, L* and h_ab_, a lower Cp indicates a greater impact. The determination of the Cp and r² were made by A520nm and CIELAB color space for each pH level (Table 1) and ethanol content (Table 2).

Table 2 presents Cp and r² values determined for model wine solutions of malvidin-3-*O*-glucoside (50 mg/L) at different pH levels supplemented with an increasing concentration of (−)-epicatechin and oenological tannins. In 72% of the cases (13/18 possibilities), supplementation with gallotannin yielded the highest hyperchromic (increasing values of A520nm and a* and decreasing values in L*) and bathochromic effects (decrease in b* and increase in C*_ab_). Significant differences were noted between the different pH levels, pH 3.1 yielding the greatest color stabilization effect for (−)-epicatechin, grape-seed and grape-skin and pH 3.5 producing that for quebracho, gallotannin and ellagitannin. In all cases, pH 3.9 was found to be the least suitable pH for the color stabilization between oenological tannins and malvidin-3-*O*-glucoside solution, although h_ab_ values were greater. In general, an increase in pH resulted in a decrease in Cp as reported for other phenolic compounds [17]. These results are also in accordance with the fact that anthocyanins are more stable at lower pH levels and thus, lower pH is favorable for color stabilization [27,28]. At pH 3.5, all tested oenological tannins, except for grape-seed, were found to induce a better color stabilization effect than (−)-epicatechin (the reference). At pH 3.1, grape-seed and quebracho tannins were weaker than (−)-epicatechin while at pH 3.9 only gallotannin and ellagitannin were more effective than (−)-epicatechin. This denotes that in general, oenological tannins present a higher capacity to stabilize the color of wines than (−)-epicatechin, which is naturally present in wine and known to be a great copigment.

### 2.3. Influence of Copigment/Pigment Ratio and Ethanol Content

Table 3 displays the Cp and r² values that were determined for different percentages of ethanol (10, 12 and 14%) in the enriched model wine solutions of malvidin-3-*O*-glucoside. Independently of ethanol content, hydrolysable tannins, especially gallotannins, provided the best color stabilization effect. These results are in accordance with those previously obtained for the pH effect. In 89% of the cases (16/18 possibilities), gallotannin yielded the highest hyperchromic and bathochromic effect and ellagitannin was the second most efficient in color stabilization. The difference in effectiveness between hydrolysable tannins and condensed were highly notable. In fact, compared to condensed tannins, gallotannin and ellagitannin reached Cp values four and two times higher, respectively. Additionally, significant differences were observed between the different ethanol contents. At 10% ethanol, the greatest color stabilization effect was observed for (−)-epicatechin and all tested oenological tannins, except for quebracho tannin. Quebracho tannin reached its maximum effect on color stabilization at 12% ethanol. Moreover, 14% ethanol appeared to be the least appropriate condition for color stabilization between oenological tannins and malvidin-3-*O*-glucoside. For all tested oenological tannins except quebracho tannin, the increase in ethanol content resulted in a decrease in the Cp. It should be highlighted that the hyperchromic effect decreased with the augmentation of the ethanol content while the bathochromic effect decreased between 10 to 12% ethanol and remained quite stable between 12 and 14% ethanol. These results are in accordance with those obtained previously [21], which demonstrated that the presence of ethanol can disrupt copigmentation complexes. Independent of ethanol content, only hydrolysable tannins were able to improve the color stabilization compared to (−)-epicatechin. Nevertheless, at 12% ethanol content, quebracho tannins presented a higher color stabilization effect than (−)-epicatechin. This means that in general, only the hydrolysable tannins present a higher capacity to stabilize the color of wines than (−)-epicatechin when the ethanol content changes.

## 3. Discussion

Oenological tannins are commercially prepared, meaning that the preparation process, extraction and raw material used will differ according to the company selling them. Thus, we can expect differences due to their complexity in tannins and to an even greater extent, differences regarding their properties. Nevertheless, previous studies have shown that chemical composition remains similar between two tannins originating from two different plant sources, but from the same family (i.e., nut galls and tara). Because each family of commercial tannins (GT, ET, PC/PD and PF/PR) presents a similar structure [12,29], it is only possible to differentiate oenological tannins according their chemical family [30]. The fact that it is difficult to distinguish commercial tannins by their plant source directly impacts their differentiation regarding their properties. Tannins from the same family present the similar effects regarding color stabilization, while tannins from different families can provide significantly different effects. Hydrolysable tannins, more specifically gallotannins, are the most effective. Regarding other properties of the oenological tannins that have been studied previously, we can make the same conclusions. For example, tannins from the same family have shown similar oxygen consumption rates and the laccase activity, while those from different chemical families do not share such properties. With respect to the antioxidant capacity, it is only possible to differentiate between condensed tannins and hydrolysable tannins, not between GT and ET or between PC/PD and PF/PR [27,29,30]. Additionally, we can expect a difference regarding the properties according to the richness in tannins of the commercial extract, but once again, this property is similar for tannins of the same chemical origin [31]. Tannins from different chemical families present differential richness as their molecular compositions change. In fact, all the results obtained lead us to conclude that it is more appropriate to classify the oenological tannins according to their botanical family than by the plant source from which they originate. It is the chemical structure of the commercial tannins rather than their plant source which dictates their properties. In addition, their chemical structure better explains the ability of some commercial tannins to consume oxygen or protect other compounds from the oxidation, and the ability of others to stabilize the color of the wines. It has been shown that gallotannins provide low oxygen consumption while they seem to be the most effective regarding the color stabilization [32].

The copigment to pigment ratio seems to also have an important impact as demonstrated in Figure 1. The increase of the Cp/p ratio induced an increase of the magnitude of the absorbance and even more so the increase of the hyperchromic and bathochromic shifts. In our experimentation, the concentration in pigment (malvidin-3-*O*-monoglucoside) was always the same (50 mg/L) meaning that the copigmentation effect depends only on the copigment concentration. Moreover, the higher the concentration of copigments (commercial tannins), the higher the changes regarding hyperchromic and bathochromic shifts. Previous studies have reported the same effects [28,33].

Complementary studies about influence of pH and ethanol content were made with five oenological tannins representing the main botanical origins used in winemaking. The first experiment allowed us to determine the most efficient oenological tannins family with respect to color improvement according to their botanical origin. Additionally, the authors desired to confirm these effects under different oenological conditions. Different pH levels (3.1, 3.5, 3.9) were tested with a constant content of ethanol (12%) and different ethanol contents (10%, 12%, 14%) were tested with a constant pH level (3.5). In fact, many authors have previously studied the impact of the pH level [34,35,36] but not so well in the presence of commercial tannins. Moreover, the impact of the ethanol content has not been well studied. The results of these studies (Figure 4, Table 2 and Table 3) confirm those previously obtained in the sense that regardless of the oenological conditions, hydrolysable tannins (gallotannin and ellagitannin) remain the most efficient oenological tannins regarding color improvement. However, the pH level and ethanol content play a certain role, since they can increase or decrease the effect of the oenological tannins as copigments. In general, high pH and ethanol content generate a diminution of the ability of commercial tannins to improve the color. This could be explained by the fact that anthocyanins are more stable at lower pH levels, allowing for greater color improvement [37]. Alcohol also works against copigmentation by destabilizing the hydrogen bonding between anthocyanin aggregates, because it can disrupt the lattice like interaction of water molecules and destroy the molecular stacking of the anthocyanins [37].

## 4. Materials and Methods

### 4.1. Chemicals and Equipments

All samples and standards were handled without any exposure to light. l-(+)-tartaric acid and sodium hydroxide were purchased from PANREAC (Barcelona, Spain). Malvidin-3-*O*-glucoside and (−)-epicatechin were purchased from Extrasynthese (Genay, France). Absolute ethanol and hydrochloric acid were supplied by Fisher Scientific (Madrid, Spain).

A spectrophotometer UV-Vis Helios Alpha™ (Thermo Fisher Scientific Inc., Waltman, MA, USA) was used for the analysis.

### 4.2. Commercial Tannins

Thirty-six commercial tannins were analyzed in this study: three tannins from grapes, four from grape seeds, two from grape skin, two from acacia, six from quebracho, four from nut galls, four from tara, eight from oak and three from chestnut. They were provided by eight different companies: Laffort (Floirac, France), Agrovin (Ciudad Real, Spain), Sofralab (Magenta, France), Institut Oenologique de Champagne (IOC) (Epernay, France), Esseco (Trecate Novara, Italy), AEB (Brescia, Italy), Erblsöh (Geisenheim, Germany) and Vason (Verona, Italy).

The richness (%) in tannins of the commercial tannins were determined in a previous study [31].

### 4.3. Influence of the Botanical Origin

The thirty-six oenological tannins were used to determine the influence of the botanical origin on the copigmentation effect. A model wine solution (12% ethanol, 4 g/L of tartaric acid and adjusted at pH 3.5) was prepared and supplemented with 0.1, 0.2 and 0.4 g/L of each of the thirty-six commercial tannins and (−)-epicatechin (copigments). The (−)-epicatechin was used as reference standard. Simultaneously, solutions containing 50 mg/L of malvidin-3-*O*-monoglucoside (pigment) and 0.1, 0.2 and 0.4 g/L of commercial tannins or (−)-epicatechin were prepared to reach copigment/pigment ratios of 2, 4 and 8 respectively. Finally, a solution containing only the malvidin was prepared as positive control. Then, 1.5 mL of each solution was placed in Eppendorf tubes and maintained under airtight conditions. A week later, the full absorbance spectrum in the visible range (400–800 nm) was measured to determine the CIELAB color space and the effectiveness of copigmentation (Cp).

### 4.4. Influence of the pH Level

Five out of the thirty-six previously tested oenological tannins (grape-seeds, grape-skins, quebracho, ellagitannins and gallotannins) representing the most common botanical origins were used to facilitate these experiments. For each oenological tannin considered, three different model wine solutions (4 g/L of tartaric acid and 12% of ethanol) were prepared with different pH levels (3.1, 3.5 and 3.9) to observe the effect of pH on color stabilization. All samples were prepared as in the previous assay (4.3). As stated previously, the samples were maintained under airtight conditions and the full absorbance spectrum was measured a week later.

### 4.5. Influence of Ethanol Content

Five out of the thirty-six previously tested oenological tannins (grape-seeds, grape-skins, quebracho, ellagitannins and gallotannins) representing the most common botanical origins were used to facilitate the experimentations. For each oenological tannin considered, three different model wine solutions (4 g/L of tartaric acid and pH adjusted at 3.5) were prepared with different ethanol contents (10, 12 and 14% ethanol) to observe the effect of ethanol on color stabilization. All the samples were prepared as in the previous assay (4.3). As stated previously, the samples were maintained in airtight conditions and the full absorbance spectrum was measured a week later.

### 4.6. Colorimetric Analysis

The full absorbance spectrum in the visible range (400–800 nm) of all samples was measured with a spectrophotometer, using a quartz cell (10 mm of path length). In all cases, the spectrum of the solution containing only the copigment (oenological tannins) was subtracted from the spectrum of the corresponding mix copigment/pigment (oenological tannins/malvidin). This subtraction was made to avoid the interferences due to the natural color of each copigment. Then, the absorbance at 520 nm (A520nm) and the CIELAB color space were calculated with an MSCV software according to Ayala et al., 1997. The comparison of the A520nm and CIELAB color space between copigment/pigment and pigment allowed us to determine the hypochromic/hyperchromic (variation of εmax) and hypsochromic/bathochromic (variation of λmax) effects of each oenological tannin.

### 4.7. Statistical Analysis

All the chemical and physical data are expressed as mean values ± standard deviation. The statistical analyses were carried out using the XLSTAT 2017 statistical package. The two hypotheses of normality and homoscedasticity of the data were tested, for all parameters, by using the Shapiro–Wilk test and Levene’s test, respectively. When populations were distributed normally and presented homogeneity in variance, parametric tests (ANOVA and Tukey) were used to detect significant differences at a 95% confidence level. In contrast, when populations were not distributed normally and/or presented heterogeneity in variance, non-parametric tests (Kruskal–Wallis and Pairwise–Wilcox) were used. Differences were statistically significant at *p*-value < 0.05.

## 5. Conclusions

These results allowed us to conclude that botanical origin of oenological tannins influence their effectiveness regarding color stabilization. Indeed, hydrolysable tannins and more specifically gallotannins are the most efficient compounds to stabilize the color of red wines during the aging. In contrast, condensed tannins seem to be weaker regarding the protection of the color even if PC/PD tannins appear to be more efficient than PF/PR tannins. Moreover, the importance of the pH level and ethanol content of the wine in presence of oenological tannins have been elucidated. Generally, the higher the pH and ethanol content, the lower the color stabilization effect. Nevertheless, the augmentation or decrease of these two parameters influence all the oenological tannins effects in the same way. Independent of the intrinsic wine conditions, hydrolysable tannins remain the most effective in stabilizing red wine color.

## Figures and Tables

**Table 1:** Copigmentation effectiveness (Cp) of model wine solution of malvidin-3-*O*-glucoside (50 mg/L) supplemented with increasing concentration of (−)-epicatechin or oenological tannins after one week of experimentation.

	Tannins	A 520		L*		a*		b*		h_ab_		C*_ab_	
	**(−)-Epicatechin**	**0.061 ± 0.008**	**b**	**−1.39 ± 0.06**	**a**	**2.29 ± 0.02**	**a**	**−1.15 ± 0.09**	**b**	**−8.9 ± 0.2**	**a**	**2.51 ± 0.03**	**a**
**PC/PD**	Grape 1	0.141 ± 0.004		-2.09 ± 0.60		2.26 ± 0.50		-1.00 ± 0.04		-7.2 ± 0.3		2.45 ± 0.05	
	Grape 2	0.145 ± 0.002		−1.47 ± 0.60		1.56 ± 0.10		−0.86 ± 0.06		−7.2 ± 0.5		1.71 ± 0.10	
	Grape 3	0.167 ± 0.002		−1.84 ± 0.00		1.93 ± 0.20		−0.88 ± 0.05		−7.5 ± 0.4		2.01 ± 0.06	
	**AV Grapes**	**0.151 ± 0.014**	**a**	**−1.80 ± 0.31**	**a**	**1.92 ± 0.35**	**a**	**−0.91 ± 0.07**	**b**	**−7.3 ± 0.2**	**a**	**2.06 ± 0.37**	**a**
	Seed 1	0.128 ± 0.001		−1.04 ± 0.00		1.06 ± 0.60		−0.47 ± 0.05		−5.2 ± 0.5		1.26 ± 0.06	
	Seed 2	0.174 ± 0.007		−2.70 ± 0.00		2.43 ± 0.50		−1.02 ± 0.04		−7.9 ± 0.5		2.49 ± 0.04	
	Seed 3	0.073 ± 0.002		−0.90 ± 0.00		1.40 ± 0.60		−0.63 ± 0.04		−6.0 ± 0.3		1.62 ± 0.04	
	Seed 4	0.054 ± 0.002		−0.63 ± 0.00		1.25 ± 0.70		−0.52 ± 0.08		−5.3 ± 0.8		1.45 ± 0.03	
	**AV Seeds**	**0.107 ± 0.055**	**a**	**−1.32 ± 0.94**	**a**	**1.54 ± 0.61**	**a**	**−0.66 ± 0.25**	**a**	**−6.1 ± 1.2**	**a**	**1.70 ± 0.54**	**a**
	Skin 1	0.195 ± 0.022		−1.96 ± 0.07		1.81 ± 0.04		−0.76 ± 0.02		−6.7 ± 0.9		2.04 ± 0.13	
	Skin 2	0.163 ± 0.002		−2.49 ± 0.07		2.37 ± 0.08		−1.40 ± 0.09		−11.3 ± 0.8		2.65 ± 0.05	
	**AV Skins**	**0.179 ± 0.022**	**a**	**−2.22 ± 0.37**	**a**	**2.09 ± 0.40**	**a**	**−1.08 ± 0.45**	**b**	**−9.0 ± 3.3**	**a**	**2.35 ± 0.43**	**a**
	**AV PC/PD**	**0.146 ± 0.036**	**B**	**−1.78 ± 0.45**	**A**	**1.85 ± 0.28**	**B**	**−0.88 ± 0.21**	**A**	**−7.5 ± 1.5**	**A**	**2.04 ± 0.32**	**C**
**PF/PR**	Acacia 1	0.125 ± 0.018		−1.41 ± 0.06		0.91 ± 0.08		−0.53 ± 0.14		−4.6 ± 0.5		1.07 ± 0.06	
	Acacia 2	0.084 ± 0.006		−1.18 ± 0.14		1.18 ± 0.07		−0.18 ± 0.09		−1.4 ± 0.8		1.20 ± 0.08	
	**AV Acacia**	**0.105 ± 0.029**	**a**	**−1.30 ± 0.16**	**a**	**1.05 ± 0.20**	**a**	**−0.36 ± 0.25**	**a**	**−3.0 ± 2.3**	**a**	**1.14 ± 0.09**	**a**
	Quebracho 1	0.078 ± 0.079		−0.97 ± 0.07		1.14 ± 0.04		−0.83 ± 0.06		−7.9 ± 0.7		1.33 ± 0.04	
	Quebracho 2	0.088 ± 0.047		−1.23 ± 0.07		1.80 ± 0.04		−2.27 ± 0.05		−8.1 ± 0.6		1.28 ± 0.11	
	Quebracho 3	0.062 ± 0.007		−1.09 ± 0.06		0.97 ± 0.13		−0.93 ± 0.05		−9.2 ± 1.6		1.39 ± 0.08	
	Quebracho 4	0.076 ± 0.004		−1.13 ± 0.06		1.13 ± 0.15		−0.99 ± 0.19		−9.6 ± 0.8		1.32 ± 0.16	
	Quebracho 5	0.118 ± 0.010		−1.49 ± 0.10		1.55 ± 0.11		−1.63 ± 0.11		−14.4 ± 0.5		1.91 ± 0.12	
	Quebracho 6	0.173 ± 0.002		−2.65 ± 0.06		2.72 ± 0.03		−2.14 ± 0.07		−16.0 ± 0.3		3.17 ± 0.03	
	**AV Quebracho**	**0.099 ± 0.041**	**a**	**−1.43 ± 0.63**	**a**	**1.55 ± 0.65**	**a**	**−1.46 ± 0.64**	**b**	**−10.9 ± 3.5**	**b**	**1.74 ± 0.74**	**a**
	**AV PF/PR**	**0.102 ± 0.004**	**B**	**−1.36 ± 0.09**	**A**	**1.30 ± 0.36**	**B**	**−0.91 ± 0.78**	**A**	**−6.96 ± 5.5**	**A**	**1.44 ± 0.42**	**C**
**GT**	Nut gall 1	0.302 ± 0.014		−2.76 ± 0.00		4.84 ± 0.09		−2.48 ± 0.10		−15.6 ± 0.1		5.33 ± 0.10	
	Nut gall 2	0.517 ± 0.029		−4.86 ± 0.21		7.01 ± 0.05		−3.78 ± 0.11		−20.0 ± 0.1		7.72 ± 0.06	
	Nut gall 3	0.234 ± 0.007		−2.78 ± 0.06		5.11 ± 0.04		−2.41 ± 0.04		−15.2 ± 0.2		5.59 ± 0.15	
	Nut gall 4	0.467 ± 0.025		-4.36 ± 0.07		6.69 ± 0.12		−4.01 ± 0.05		−23.5 ± 0.1		7.55 ± 0.13	
	**AV Nut galls**	**0.380 ± 0.134**	**a**	**−3.69 ± 1.08**	**a**	**5.91 ± 1.10**	**a**	**−3.17 ± 0.84**	**a**	**−18.6 ± 3.9**	**a**	**6.55 ± 1.26**	**a**
	Tara 1	0.520 ± 0.047		−4.94 ± 0.07		7.38 ± 0.03		−4.21 ± 0.07		−14.9 ± 0.1		5.61 ± 0.03	
	Tara 2	0.409 ± 0.009		−5.18 ± 0.06		7.42 ± 0.07		−4.29 ± 0.06		−12.9 ± 0.1		4.61 ± 0.06	
	Tara 3	0.588 ± 0.008		−5.45 ± 0.06		8.44 ± 0.07		−5.65 ± 0.04		−16.4 ± 0.1		5.82 ± 0.09	
	Tara 4	0.512 ± 0.004		−4.90 ± 0.06		7.41 ± 0.06		−4.22 ± 0.06		−14.8 ± 0.2		8.59 ± 0.06	
	**AV Tara**	**0.507 ± 0.074**	**a**	**−5.12 ± 0.25**	**b**	**7.66 ± 0.52**	**a**	**−4.59 ± 0.71**	**a**	**−14.7 ± 1.4**	**a**	**6.16 ± 1.71**	**a**
	**AV GT**	**0.443 ± 0.121**	**A**	**−4.40 ± 1.06**	**B**	**6.79 ± 1.23**	**A**	**−3.88 ± 1.05**	**B**	**−16.7 ± 3.4**	**B**	**6.35 ± 1.41**	**A**
**ET**	Chestnut 1	0.400 ± 0.012		−4.03 ± 0.07		4.06 ± 0.09		−3.48 ± 0.06		−22.8 ± 0.1		4.79 ± 0.09	
	Chestnut 2	0.219 ± 0.005		−3.48 ± 0.12		3.96 ± 0.04		−3.46 ± 0.02		−23.5 ± 0.1		4.72 ± 0.13	
	Chestnut 3	0.219 ± 0.009		−3.54 ± 0.10		4.13 ± 0.08		−3.48 ± 0.06		−23.3 ± 0.2		4.88 ± 0.08	
	**AV Chestnut**	**0.279 ± 0.105**	**a**	**−3.68 ± 0.30**	**a**	**4.05 ± 0.08**	**a**	**−3.47 ± 0.01**	**a**	**−23.2 ± 0.4**	**a**	**4.80 ± 0.08**	**a**
	Oak 1	0.222 ± 0.007		−3.06 ± 0.07		5.98 ± 0.10		−2.19 ± 0.08		−15.3 ± 0.1		3.83 ± 0.11	
	Oak 2	0.307 ± 0.089		−3.66 ± 0.07		6.35 ± 0.09		−5.32 ± 0.09		−36.8 ± 0.0		5.28 ± 0.08	
	Oak 3	0.212 ± 0.014		−2.79 ± 0.00		5.28 ± 0.10		−2.18 ± 0.03		−22.4 ± 0.1		3.03 ± 0.10	
	Oak 4	0.290 ± 0.036		−3.91 ± 0.21		3.89 ± 0.13		−6.16 ± 0.07		−27.9 ± 0.1		4.27 ± 0.08	
	Oak 5	0.173 ± 0.018		−2.44 ± 0.12		5.08 ± 0.08		−2.30 ± 0.07		−17.9 ± 0.1		2.73 ± 0.13	
	Oak 6	0.262 ± 0.008		−2.76 ± 0.00		3.09 ± 0.05		−2.32 ± 0.08		−11.2 ± 0.1		2.57 ± 0.13	
	Oak 7	0.272 ± 0.006		−2.91 ± 0.07		4.00 ± 0.11		−3.18 ± 0.04		−10.0 ± 0.1		2.78 ± 0.07	
	Oak 8	0.353 ± 0.090		−2.96 ± 0.07		5.38 ± 0.08		−2.41 ± 0.04		−10.2 ± 0.1		2.86 ± 0.12	
	**AV Oak**	**0.261 ± 0.058**	**a**	**−3.06 ± 0.49**	**a**	**4.88 ± 1.12**	**a**	**−3.26 ± 1.58**	**a**	**−19.0 ± 9.6**	**a**	**3.42 ± 0.96**	**a**
	**AV ET**	**0.270 ± 0.068**	**AB**	**−3.37 ± 0.52**	**B**	**4.46 ± 1.02**	**A**	**−3.37 ± 1.33**	**B**	**−21.1 ± 8.2**	**B**	**4.11 ± 1.03**	**B**

All data are the mean ± SD of three replicates. Stats: Statistic. Different low caster letters indicate the existence of significant differences between tannins of different botanical origin in the same family (*p* < 0.05). PC/PD: Procyanidins/Prodelphinidins; PF/PR: Profisetinidins/Prorobitenidins; GT: Gallotannins; ET: Ellagitannins; AV: Average.

**Table 2:** Copigmentation effectiveness (Cp) of model wine solutions of malvidin-3-*O*-glucoside (50 mg/L) at different pH levels, supplemented with increasing concentration of (−)-epicatechin and oenological tannins after one week of experimentation.

		A 520				L*				C*_ab_				h_ab_				a*				b*			
	**Tanins**	**Cp**	**r²**	**Stats**		**Cp**	**r²**	**Stats**		**Cp**	**r²**	**Stats**		**Cp**	**r²**	**Stats**		**Cp**	**r²**	**Stats**		**Cp**	**r²**	**Stats**	
**pH 3.1**	**(−)-Epicatechin**	0.12 ± 0.00	0.9997	**C**	**α**	−1.68 ± 0.00	0.9881	**C**	**β**	2.40 ± 0.02	0.9978	**D**	**α**	−5.50 ± 0.00	0.9985	**AB**	**β**	2.19 ± 0.02	0.9976	**AB**	**α**	−1.53 ± 0.02	0.9828	**A**	**β**
	**Grape−seed**	0.11 ± 0.00	0.9908	**D**	**α**	−1.55 ± 0.01	0.9993	**B**	**β**	1.95 ± 0.01	0.9925	**E**	**α**	−6.95 ± 0.00	0.9987	**AB**	**αβ**	1.70 ± 0.01	0.9925	**AB**	**α**	−1.70 ± 0.04	0.9842	**AB**	**β**
	**Grape−skin**	0.25 ± 0.01	0.9768	**A**	**α**	−3.07 ± 0.00	0.9856	**F**	**β**	3.97 ± 0.00	0.9807	**B**	**α**	−4.96 ± 0.00	0.9750	**B**	**α**	3.71 ± 0.00	0.9779	**AB**	**α**	−2.07 ± 0.02	0.9989	**AB**	**β**
	**Quebracho**	0.02 ± 0.00	0.8904	**E**	**χ**	−0.92 ± 0.00	0.9758	**A**	**α**	0.13 ± 0.04	0.7953	**F**	**β**	−7.87 ± 0.00	0.9946	**AB**	**α**	−0.16 ± 0.03	0.8585	**B**	**β**	−1.61 ± 0.04	0.9933	**AB**	**αβ**
	**Gallotannin**	0.22 ± 0.00	0.8446	**B**	**β**	−3.02 ± 0.00	0.8318	**E**	**αβ**	4.49 ± 0.03	0.8377	**A**	**αβ**	−5.36 ± 0.00	0.9173	**AB**	**α**	3.98 ± 0.02	0.8384	**A**	**αβ**	−3.18 ± 0.04	0.9953	**B**	**αβ**
	**Ellagitannin**	0.13 ± 0.00	0.8334	**C**	**β**	−2.71 ± 0.00	0.8824	**D**	**β**	2.52 ± 0.01	0.8371	**C**	**α**	−13.05 ± 0.00	0.9955	**A**	**αβ**	2.00 ± 0.01	0.8233	**AB**	**αβ**	−3.09 ± 0.02	0.9952	**AB**	**β**
**pH 3.5**	**(−)−Epicatechin**	0.06 ± 0.00	0.8940	**E**	**χ**	−0.92 ± 0.00	0.9231	**A**	**α**	1.20 ± 0.03	0.8937	**D**	**β**	−4.06 ± 0.00	0.9763	**B**	**αβ**	1.12 ± 0.03	0.8744	**A**	**β**	−0.62 ± 0.03	1.0000	**AB**	**αβ**
	**Grape−seed**	0.07 ± 0.00	0.9681	**E**	**β**	−1.00 ± 0.00	1.0000	**B**	**αβ**	1.28 ± 0.02	0.9619	**D**	**αβ**	−3.01 ± 0.01	0.9776	**AB**	**α**	1.23 ± 0.02	0.9702	**AB**	**αβ**	−0.52 ± 0.04	0.8378	**A**	**α**
	**Grape−skin**	0.12 ± 0.00	0.9581	**D**	**β**	−1.91 ± 0.00	0.9791	**C**	**αβ**	2.01 ± 0.06	0.9660	**C**	**αβ**	−7.74 ± 0.01	0.9617	**C**	**αβ**	1.83 ± 0.05	0.9610	**AB**	**αβ**	−1.22 ± 0.06	0.8009	**AB**	**αβ**
	**Quebracho**	0.17 ± 0.00	0.9251	**B**	**α**	−2.33 ± 0.00	0.9018	**D**	**β**	2.50 ± 0.06	0.9525	**B**	**α**	−8.74 ± 0.01	0.9995	**D**	**αβ**	2.38 ± 0.06	0.9562	**AB**	**α**	−1.15 ± 0.08	0.7987	**AB**	**α**
	**Gallotannin**	0.31 ± 0.00	0.9885	**A**	**α**	−4.14 ± 0.00	0.9832	**F**	**β**	6.23 ± 0.03	0.9897	**A**	**α**	−16.75 ± 0.01	0.9516	**F**	**β**	5.62 ± 0.02	0.0885	**B**	**α**	−3.41 ± 0.05	0.9986	**B**	**β**
	**Ellagitannin**	0.15 ± 0.00	0.9964	**C**	**α**	−2.48 ± 0.00	0.9976	**E**	**αβ**	2.52 ± 0.02	0.9959	**B**	**α**	−12.74 ± 0.00	0.9828	**E**	**α**	2.19 ± 0.01	0.9930	**AB**	**α**	−1.90 ± 0.01	0.9998	**AB**	**αβ**
**pH 3.9**	**(−)-Epicatechin**	0.08 ± 0.00	1.0000	**C**	**β**	−1.08 ± 0.00	0.9826	**C**	**αβ**	1.26 ± 0.01	0.9922	**C**	**αβ**	−3.79 ± 0.00	0.9932	**A**	**α**	1.20 ± 0.01	0.9908	**AB**	**αβ**	−0.45 ± 0.02	0.9967	**AB**	**α**
	**Grape-seed**	0.05 ± 0.00	0.9873	**D**	**χ**	−0.79 ± 0.00	0.9973	**A**	**α**	0.71 ± 0.01	0.9304	**F**	**β**	−7.63 ± 0.07	1.0000	**B**	**β**	0.61 ± 0.02	0.9131	**B**	**β**	−0.62 ± 0.07	0.8536	**AB**	**αβ**
	**Grape-skin**	0.06 ± 0.01	0.9873	**D**	**χ**	−0.92 ± 0.00	0.8811	**B**	**α**	0.85 ± 0.02	0.9996	**E**	**β**	−11.63 ± 0.01	0.9001	**C**	**β**	0.70 ± 0.01	0.7340	**AB**	**β**	−0.15 ± 0.13	0.9251	**A**	**α**
	**Quebracho**	0.05 ± 0.00	0.9434	**D**	**β**	−1.75 ± 0.00	0.9643	**E**	**αβ**	0.98 ± 0.03	0.8275	**D**	**αβ**	−11.92 ± 0.01	0.9826	**D**	**β**	0.80 ± 0.03	0.9943	**AB**	**αβ**	−3.07 ± 0.05	0.9588	**B**	**β**
	**Gallotannin**	0.15 ± 0.00	0.9920	**A**	**χ**	−2.08 ± 0.00	0.9952	**F**	**α**	3.04 ± 0.03	0.9954	**A**	**β**	−16.14 ± 0.02	0.9924	**E**	**αβ**	2.74 ± 0.02	0.9922	**A**	**β**	−1.75 ± 0.05	0.9969	**AB**	**α**
	**Ellagitannin**	0.10 ± 0.00	0.9510	**B**	**χ**	−1.65 ± 0.00	0.9443	**D**	**α**	1.62 ± 0.01	0.9615	**B**	**α**	−18.34 ± 0.02	0.9968	**F**	**β**	1.33 ± 0.01	0.9692	**AB**	**β**	−1.51 ± 0.07	0.9898	**AB**	**α**

All data are the mean ± SD of three replicates. Stats: Statistic. Different capital caster letters indicate the existence of significant differences between tannins for the same pH level (*p* < 0.05). Different Greek letters indicates the existence of significant differences between pH level for the same tannin (*p* < 0.05).

**Table 3:** Copigmentation effectiveness (Cp) of model wine solutions of malvidin-3-*O*-glucoside (50 mg/L) at different ethanol (EtOH) contents, supplemented with increasing concentrations of (−)-epicatechin and oenological tannins after one week of experimentation.

		A 520				L*				C*_ab_				h_ab_				a*				b*			
	**Tanins**	**Cp**	**r²**	**Stats**		**Cp**	**r²**	**Stats**		**Cp**	**r²**	**Stats**		**Cp**	**r²**	**Stats**		**Cp**	**r²**	**Stats**		**Cp**	**r²**	**Stats**	
**EtOH 10**	**(−)-Epicatechin**	0.15 ± 0.00	0.9999	**C**	**α**	−1.75 ± 0.00	0.9993	**B**	**β**	2.77 ± 0.02	0.9989	**C**	**α**	−7.02 ± 0.01	0.9717	**B**	**β**	2.62 ± 0.02	0.9980	**C**	**α**	−1.19 ± 0.04	0.9828	**C**	**β**
	**Grape-seed**	0.12 ± 0.00	0.9991	**D**	**α**	−1.46 ± 0.00	0.9909	**A**	**β**	1.83 ± 0.03	0.9983	**E**	**α**	−6.27 ± 0.01	0.9777	**A**	**αβ**	1.71 ± 0.02	0.9984	**E**	**α**	−0.94 ± 0.04	0.9842	**A**	**αβ**
	**Grape-skin**	0.14 ± 0.00	0.9849	**C**	**α**	−2.11 ± 0.00	0.9861	**D**	**β**	1.97 ± 0.01	0.9827	**D**	**α**	−8.50 ± 0.01	0.9967	**C**	**β**	2.15 ± 0.01	0.9857	**D**	**α**	−1.27 ± 0.04	0.9989	**B**	**β**
	**Quebracho**	0.12 ± 0.01	0.9775	**D**	**αβ**	−2.03 ± 0.00	0.9798	**C**	**αβ**	1.09 ± 0.05	0.8761	**F**	**α**	−14.97 ± 0.02	0.9875	**D**	**β**	1.53 ± 0.05	0.9521	**F**	**α**	−1.94 ± 0.13	0.9933	**C**	**β**
	**Gallotannin**	0.47 ± 0.00	0.9983	**A**	**α**	−6.30 ± 0.00	0.9979	**F**	**β**	8.33 ± 0.02	0.9979	**A**	**α**	−25.68 ± 0.01	0.9424	**F**	**β**	9.39 ± 0.02	0.9983	**A**	**α**	−5.49 ± 0.07	0.9953	**E**	**β**
	**Ellagitannin**	0.23 ± 0.00	0.9983	**B**	**α**	−3.48 ± 0.00	0.9996	**E**	**β**	4.43 ± 0.02	0.9858	**B**	**α**	−20.82 ± 0.00	0.9993	**E**	**β**	3.82 ± 0.01	0.9871	**B**	**α**	−3.31 ± 0.02	0.9952	**D**	**α**
**EtOH 12**	**(−)-Epicatechin**	0.10 ± 0.00	0.9845	**D**	**αβ**	−1.42 ± 0.00	0.9935	**B**	**αβ**	1.78 ± 0.16	0.9267	**C**	**α**	−3.55 ± 0.01	0.9705	**A**	**α**	1.93 ± 0.11	0.9778	**B**	**αβ**	−0.73 ± 0.12	1.0000	**C**	**α**
	**Grape-seed**	0.07 ± 0.00	0.9902	**F**	**αβ**	−0.56 ± 0.00	0.3213	**A**	**α**	0.49 ± 0.09	0.5663	**E**	**β**	−5.53 ± 0.02	0.8155	**C**	**α**	0.30 ± 0.06	0.4808	**D**	**β**	0.64 ± 0.17	0.8378	**B**	**α**
	**Grape-skin**	0.08 ± 0.00	0.8542	**E**	**β**	−1.59 ± 0.00	0.9498	**C**	**αβ**	0.87 ± 0.05	0.7500	**DE**	**β**	−5.26 ± 0.04	0.7940	**B**	**α**	1.35 ± 0.05	0.9959	**C**	**αβ**	−0.63 ± 0.07	0.8009	**C**	**α**
	**Quebracho**	0.22 ± 0.00	0.9554	**B**	**α**	−3.24 ± 0.00	0.9672	**E**	**β**	0.98 ± 0.27	0.9600	**D**	**α**	−9.77 ± 0.02	0.7591	**D**	**αβ**	1.10 ± 0.19	0.9568	**C**	**αβ**	6.46 ± 0.12	0.7987	**A**	**α**
	**Gallotannin**	0.38 ± 0.00	0.9999	**A**	**αβ**	−5.22 ± 0.00	0.9994	**F**	**αβ**	6.88 ± 0.04	0.9945	**A**	**α**	−19.07 ± 0.02	0.9829	**F**	**α**	6.39 ± 0.08	0.9990	**A**	**αβ**	−4.09 ± 0.09	0.9986	**E**	**α**
	**Ellagitannin**	0.16 ± 0.00	0.9893	**C**	**αβ**	−2.72 ± 0.00	0.9840	**D**	**αβ**	2.80 ± 0.14	0.9932	**B**	**α**	−16.45 ± 0.07	1.0000	**E**	**αβ**	2.05 ± 0.10	0.9590	**B**	**β**	−2.25 ± 0.46	0.9998	**D**	**α**
**EtOH 14**	**(−)-Epicatechin**	0.09 ± 0.00	0.9980	**C**	**β**	−1.17 ± 0.00	0.9937	**B**	**α**	1.75 ± 0.02	0.9977	**C**	**α**	−6.97 ± 0.00	0.9963	**B**	**αβ**	1.60 ± 0.02	0.9972	**C**	**β**	−1.08 ± 0.02	0.9967	**B**	**αβ**
	**Grape-seed**	0.06 ± 0.00	0.9954	**E**	**β**	−0.83 ± 0.00	0.9868	**A**	**αβ**	1.52 ± 0.02	0.9834	**D**	**αβ**	−35.41 ± 0.00	0.8453	**F**	**β**	1.02 ± 0.02	0.9999	**E**	**αβ**	−4.28 ± 0.03	0.8536	**D**	**β**
	**Grape-skin**	0.09 ± 0.00	0.9133	**C**	**αβ**	−1.44 ± 0.00	0.9651	**D**	**α**	1.32 ± 0.01	0.8271	**E**	**αβ**	−5.70 ± 0.02	0.9218	**A**	**αβ**	1.20 ± 0.01	0.8127	**D**	**β**	−0.87 ± 0.06	0.9251	**A**	**αβ**
	**Quebracho**	0.06 ± 0.00	0.7889	**D**	**β**	−1.35 ± 0.00	0.7311	**C**	**α**	1.07 ± 0.03	0.9791	**F**	**α**	−9.21 ± 0.01	0.9875	**C**	**α**	0.87 ± 0.02	0.9530	**F**	**β**	−1.14 ± 0.04	0.9588	**B**	**αβ**
	**Gallotannin**	0.34 ± 0.00	0.9982	**A**	**β**	−4.52 ± 0.00	0.9993	**F**	**α**	6.91 ± 0.03	0.9981	**A**	**α**	−20.86 ± 0.02	0.9644	**E**	**αβ**	6.11 ± 0.03	0.9974	**A**	**β**	−4.23 ± 0.08	0.9969	**D**	**αβ**
	**Ellagitannin**	0.14 ± 0.00	0.9328	**B**	**β**	−2.23 ± 0.00	0.9516	**E**	**α**	2.89 ± 0.01	0.9764	**B**	**α**	−16.15 ± 0.03	0.9745	**D**	**α**	2.44 ± 0.00	0.9680	**B**	**αβ**	−2.47 ± 0.10	0.9898	**C**	**α**

All data are the mean ± SD of three replicates. Stats: Statistic. Different capital caster letters indicate the existence of significant differences between tannins for the same ethanol content (*p* < 0.05). Different Greek letters indicates the existence of significant differences between ethanol content for the same tannin (*p* < 0.05).
